# Elevated expression of miR-146a correlates with high levels of immune cell exhaustion markers and suppresses cellular immune function in chronic HIV-1-infected patients

**DOI:** 10.1038/s41598-019-55100-2

**Published:** 2019-12-11

**Authors:** Ting Yu, Zhao Ju, Mingqi Luo, Ronghua Hu, Yan Teng, Linlin Xie, Chaojie Zhong, Lang Chen, Wei Hou, Yong Xiong, Yong Feng

**Affiliations:** 1 State Key Laboratory of Virology/Institute of Medical Virology/Hubei Province Key Laboratory of Allergy & Immunology, School of Basic Medical Sciences, Wuhan University, Wuhan, 430071 Hubei People’s Republic of China; 2 Department of Infectious Diseases, Zhongnan Hospital of Wuhan University, Wuhan, 430071 Hubei People’s Republic of China; 3 Precision Medicine Laboratory, Wuhan Children’s Hospital, Wuhan, 430015 Hubei People’s Republic of China

**Keywords:** Retrovirus, Infection

## Abstract

Functional exhaustion of immune cells is a defining characteristic of HIV-1 chronic infections, exhibiting dysregulation of cellular immune responses and expression of co-inhibitory receptors. Although the molecular mechanisms controlling immune-cell exhaustion retains largely unknown, immune checkpoint blockade strategy has shown inspiring potential to reinvigorate T cell functions in chronic infections. In this study, we investigated peripheral blood mononuclear cells (PBMCs) exhaustion markers from 109 chronic HIV-1-infected patients and found they correlated positively with microRNA-146a, which was inversely correlated with CD4+ T cell count. Intriguingly, *ex vivo* neutralization of miR-146a in PBMCs from chronic HIV-1 infection exhibited an elevated antiviral cytokines production as well as the expression of GZMB and perforin, while simultaneously, decreased the inhibitory receptors expression such as PD-1, CTLA-4, TIM-3 and LAG-3. These results highlight the importance of miR-146a to HIV-1 induced immune cell exhaustion, and uncover a novel layer of HIV/AIDS pathogenesis and provide potential targets for improved immune intervention.

## Introduction

In acute infections, immune negative regulation mechanisms, such as co-inhibitory receptors cascade, function to dampen the magnitude of immune responses, which eventually decrease after pathogen clearance to achieve homeostasis. However, this pattern diverges during chronic infections, where higher and sustained expression of co-inhibitory receptors lead to dysregulation of cellular immune responses, which overtaxes the exhausted immune system. But these chronic infection-hijacked-immune negative regulation patterns provide therapeutic targets to restore optimum immune responses by check-point molecules based therapy^1^. Great success of these immune therapy in anti-tumor application raised the attempt in the control of chronic infection. In human immunodeficiency virus type 1 (HIV-1)-infected patients, PD-1 expression on virus-specific T cells is associated with clinical and virological outcomes while anti-PD-1/PD-L1 enhances HIV-1 specific T-cell function *in vitro*^2,3^ and *in vivo*^4^, as well as in SIV-infected macaques^5^. These trials suggest attractive potential to use immune checkpoint-based strategy in treating chronic infections.

Other strategies such as chimeric antigen receptor (CAR) T cells were applied to overcome T cell exhaustion in cancer^6^ and chronic infections^7^. Combinations of antiretroviral therapy (ART) with type-1 interferon (IFN-I) has also yielded positive results by reducing the viral loads and restoring CD8+ T-cell functions^8^. Recently, a combined inhibition of PD-1 and miR-146a showed attractive potential to enhance antitumor immune response elicited by checkpoint therapy^9^. Therefore, identifying new regulators of immune exhaustion upon HIV-1 infection is crucial for a comprehensive understanding of HIV/AIDS pathogenesis and development of new therapeutic strategies to improve the efficacy of immune therapy.

Micro-RNAs (miRNAs) are small non-coding RNAs that regulating gene expression at the post-transcriptional level via partial complementation to target gene’s 3’ UTR (untranslated regions)^10^. This partial complementation to target seed sequences provides a micro-RNA potential to regulate a set of target genes. Previous reports showed increasing miR-146a expression during HIV-1 infection^11,12^. By targeting TNF receptor associated factor 6 (TRAF6) and Interleukin-1 receptor-associated kinase 1 (IRAK1), miR-146a was well-documented in diverse regulatory aspects of immune responses, including its critical role in the suppressor function of Treg cells^13^ and the regulation of T cell activation^14,15^ as well as inflammatory process^16^. Moreover, miR-146a overexpression impairs the positive selection during T cell development^17^, silencing miR-146a results in functional defects of B cells^18^, and miR-146a negatively regulates NK cell functions^19^. Several studies have found miR-146a also involved in macrophage, DC function, as well as monocytes migration^20–23^. While the crucial role of miR-146a-mediated signaling pathways in host cellular immunity is widely defined, the mechanisms for the regulation of miR-146a-triggered immune exhaustion in chronic HIV-1 infection are largely unknown.

In this study, we investigated the expression levels of miR-146a in PBMCs from chronic HIV-1-infected patients and evaluated the correlation between miR-146a and immune exhaustion markers. We also tried to restore cellular immune function and reverse the exhaustion state by neutralization of miR-146a in chronic infected PBMCs *ex vivo*.

## Materials and Methods

### Study subjects

HIV-1-infected patients were recruited from Zhongnan Hospital of Wuhan University. HIV-1 infection was diagnosed on the basis of positive results in the serological and HIV RNA detection assays. The demographic and clinical characteristics of the enrolled subjects are listed in Table 1. Blood samples from 35 apparently healthy uninfected control subjects (gender- and age-matched individuals) were also participated in this study to compare the expression of miR-146a in naive HIV-1 positive patients and healthy subjects. This study received approval from the Ethical Committee, Zhongnan Hospital of Wuhan University (Ethical approval #2018006), and all research was performed in accordance with relevant guidelines. Written informed consent was obtained from each subject.

**Table 1:** The clinical characteristics of studied subjects.

Group	HCs	Early stage	Chronic HIV-1	
			CD4 < 350	CD4 ≥ 350
Cases	35	96	77	32
Age (range)	40(23–58)	45(19–80)	45 (22–78)	45 (24–70)
Gender (male/female)	21/14	85/11	54/23	17/15
CD4+ T lymphocyte count, cells/μl	ND	104.36	184.84	595
Untreated for HIV-1, n (%)	ND	73(76.0%)	18(23.4%)	3(9.38%)
Duration of HIV-1 infection (years)	ND	<0.5	0.5–13	0.5–16

HC, Healthy control; ND, no data.

### PBMC isolation

HIV-1-infected patients and healthy donors were recruited from Zhongnan Hospital of Wuhan University. Peripheral blood was centrifuged with Lymphoprep (Axis-Shield, USA) to separate the human peripheral blood mononuclear cells (PBMCs).

### CD8+ T lymphocytes isolation

CD8+ T lymphocytes were isolated from PBMC collected from healthy individuals by negative selection using the MACS® Technology, according to the manufacturer’s protocol.

### MicroRNA-146a overexpression or inhibition

PBMCs or CD8+ T lymphocytes were transfected using INTERFERin (Polyplus-Transfection, NY) following the manufacturer’s protocol with 50 nmol/ml of miR-146a mimic or miR-146a inhibitor in OptiMEM medium. The miR-146a mimic (dsRNA oligos), miRNA mimic control (mNC), inhibitor of miR-146a, and iNC were ordered from Ribobio (Guangzhou, China). Briefly, 5 × 10^6^ cells were transfected with oligonucleotides (50 nM/10^6^ cells) and then cultured for two days prior to detection.

### RNA isolation, reverse transcription, and realtime quantitative PCR

Total RNA from cultured cells was extracted with TRIzol (Invitrogen, USA) and reverse-transcribed into cDNA using the M-MLV (Promega, USA) in a total volume of 20 µl. Realtime PCR was performed using a master mix for SYBR Green qPCR (Bio-Rad Laboratories, USA) in a CFX96 Real-Time System. The reaction mix included 10 µl SYBR Green Master Mix, 0.3 µl each of forward and reverse primers, 2 µl cDNA, and was taken to a final volume of 20 µl with water. The primers for detecting the mRNA of HIV-1 Gag, PD-1, CTLA-4, TIM-3, LAG-3, GZMB, Perforin, CD107a, IL-2, TNF-α, IFN-γ, c-Fos and GAPDH were purchased from TsingKe Biological Technology (Wuhan, China). We calculated the relative expression level of each gene as the 2−ΔΔt method.

### Detection of miR-146a in peripheral blood

Each participant was collected about 5 ml of whole blood containing EDTA. Total RNA was extracted from PBMCs using TRIzol reagent (Invitrogen, Carlsbad, CA, USA), according to the manufacturer’s protocol. The quantitative analysis of miR-146a was performed using real-time quantitative reverse transcription polymerase chain reaction (qRT-PCR) with a Bulge-LoopTM miRNA qRT-PCR Starter Kit and an hsa-miR-146a qRT-PCR primer set (Ribobio, Guangzhou, China). A U6 small nuclear RNA (snRNA) primer set (Ribobio) was used as an internal control. The experiments were performed according to the protocol provided in the kit using a 20 µl reaction system.

### Detection of cytokines by ELISA

ELISA tests for detection of IFN-γ, IL-2, and TNF-α (4A Biotech, Beijing, China) in serum or supernatants of cell culture were performed using ELISA kits. The supernatant samples were diluted two-fold and the kits were applied according to

the instructions. The cytokine levels were determined from the standard curve, which was generated by the results of the standard samples provided by the manufacturers.

### Western blot

40 µg of protein from each lysate was separated by SDS-PAGE and transferred into a PVDF membrane (Millipore, USA). After blocking with 5% nonfat dry milk in 50 mM Tris-HCl (pH 7.6), 150 mM NaCl and 0.1% Tween 20, 1–2 mg/ml of anti-c-Fos primary antibody (cat# 66590-1-lg, Proteintech Group, Inc) was added and incubated overnight at 4 °C. PVDF membrane was then washed and incubated with horseradish peroxidase-labeled secondary antibodies for 2 h, followed by treatment of the membrane with the ECL reagent (Millipore, USA) and imaging on Fujifilm LAS 4000 membrane. GAPDH monoclonal antibody (Tianjin Sungene Biotech, Beijing, China) was used as an internal reference.

### Statistical analysis

Every experiment was repeated three times with duplicates, and data are reported as the mean ± SD. Statistical analyses were performed using SPSS 16.0 (SPSS Inc., Chicago, USA). A Student’s t-test was used to compare two groups, and a one-way analysis of variance (ANOVA) was used when comparing three or more groups. Correlations were analyzed using Spearman’s correlation test. Statistical significance was set at a level of p less than 0.05.

## Results

### PBMCs from Chronic HIV-1-infected patients exhibited an exhausting state

**Figure 1:**
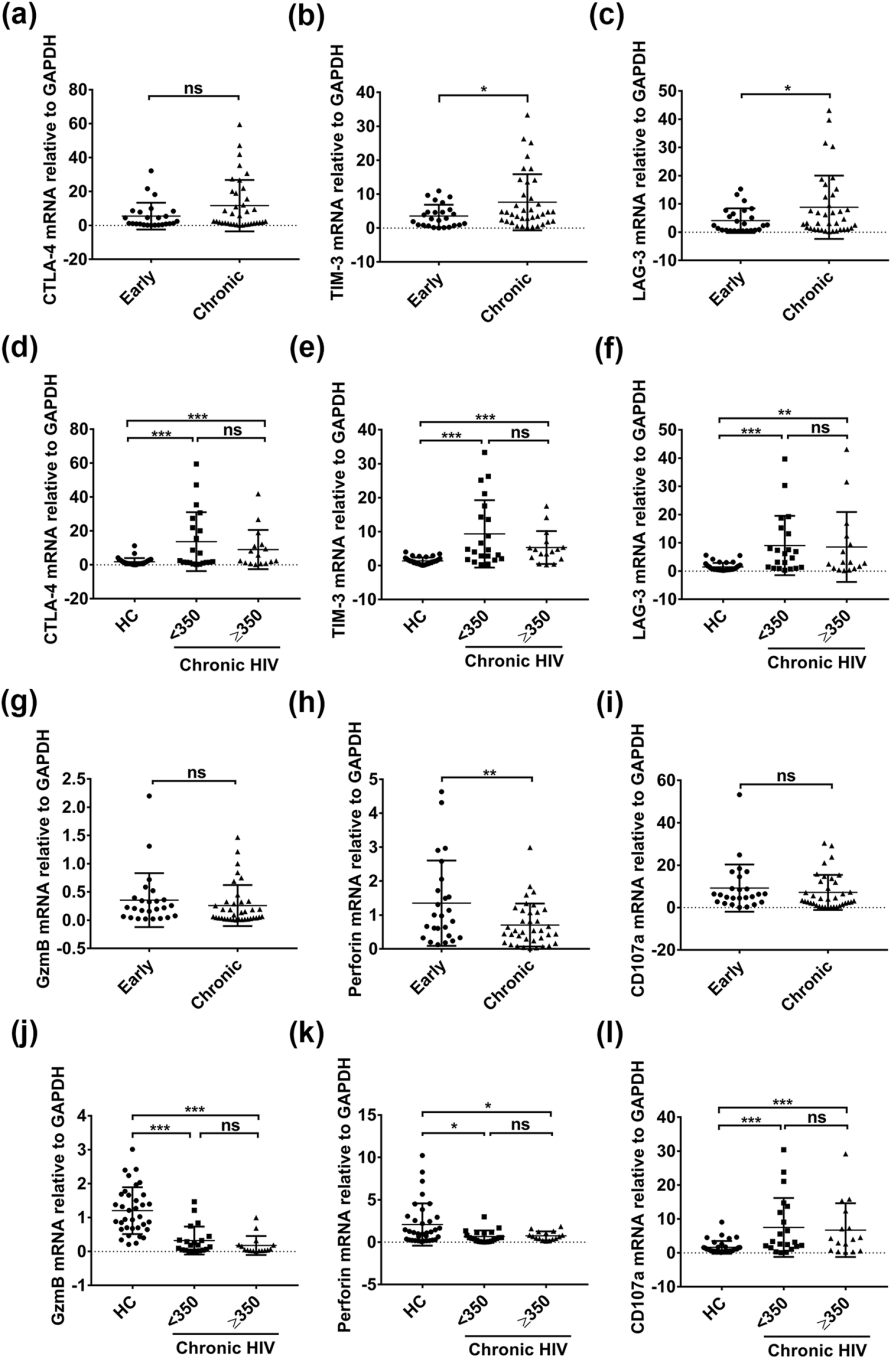
Exhaustion markers and effector function related genes expression of PBMCs from HIV-1 infected and uninfected individuals. RNA were isolated from patients’ PBMCs and analyzed for CTLA-4, TIM-3, LAG-3, GZMB, Perforin and CD107a mRNA levels by quantitative RT-PCR. Total of 205 patients’ samples were analyzed in this study, convincing data of 25 early stage and 37 chronic stage individuals were shown (**a**–**c**,**g**–**i**). mRNA levels in CD4+ T cell counts < 350 cells/µl group (n = 21) or CD4+ T cell counts ≥350 cells/µl group (n = 16) from chronic stage individuals were compared with healthy controls (HCs) (n = 35) (**d**–**f**,**j**–**l**). GAPDH was used as an endogenous control. *p < 0.05, **p < 0.01, ***p < 0.001. ns, not significant.

Besides most widely studied exhausting of CD8+ T cell, other types of immune cells, such as B cells and NK cells as well exist exhaustion during chronic HIV-1 infection. To this context, we first compared the mRNA levels of CTLA-4, TIM-3 and LAG-3, termed exhaustion markers, in PBMCs from 35 chronic HIV-1-infected patients (infected more than 6 months) with 27 patients in early stage group (infected within 6 months). In order to compare genes expression of exhaustion markers versus miR-146a, we detected mRNA levels of these genes by RT-qPCR instead of by Flow Cytometry in this study. Consistent with previous reports, exhaustion markers TIM-3 and LAG-3 were higher in chronic group (Fig. 1a–c). Levels of CTLA-4, TIM-3 and LAG-3 were also higher in PBMCs from chronic HIV-1-infected patients as compared with noninfected individuals (Healthy Control, HC) (p < 0.01) (Fig. 1d–f). There was no statistically significant difference between CD4+ T cell count under or above 350 cells/µl groups. We failed to detect PD-1 mRNAs in these samples due to abnormal higher CT value during RT-qPCR.

Next, we detected the mRNA levels of GZMB, perforin and CD107a in PBMCs, which are closely associated with T cell function. GZMB levels were lower in chronic HIV-1 group than HC group, though there was no difference between Chronic and Early groups (Fig. 1g,j). Perforin levels were lower in Chronic stage as compared with Early stage group, and were also lower than HC (Fig. 1h,k). However CD107a showed a higher level in chronic infection group as compared with HC (p < 0.01) (Fig. 1l). Notably, levels of GZMB, perforin and CD107a did not differ when grouped upon CD4+ T cell counts (Fig. 1j–l).

Taken together, these results revealed that PBMCs from chronic HIV-1-infected patients exhibited an exhausting state.

### Peripheral blood of chronic HIV-1-infected patients had higher miR-146a levels which were positively associated with immune exhaustion markers

**Figure 2:**
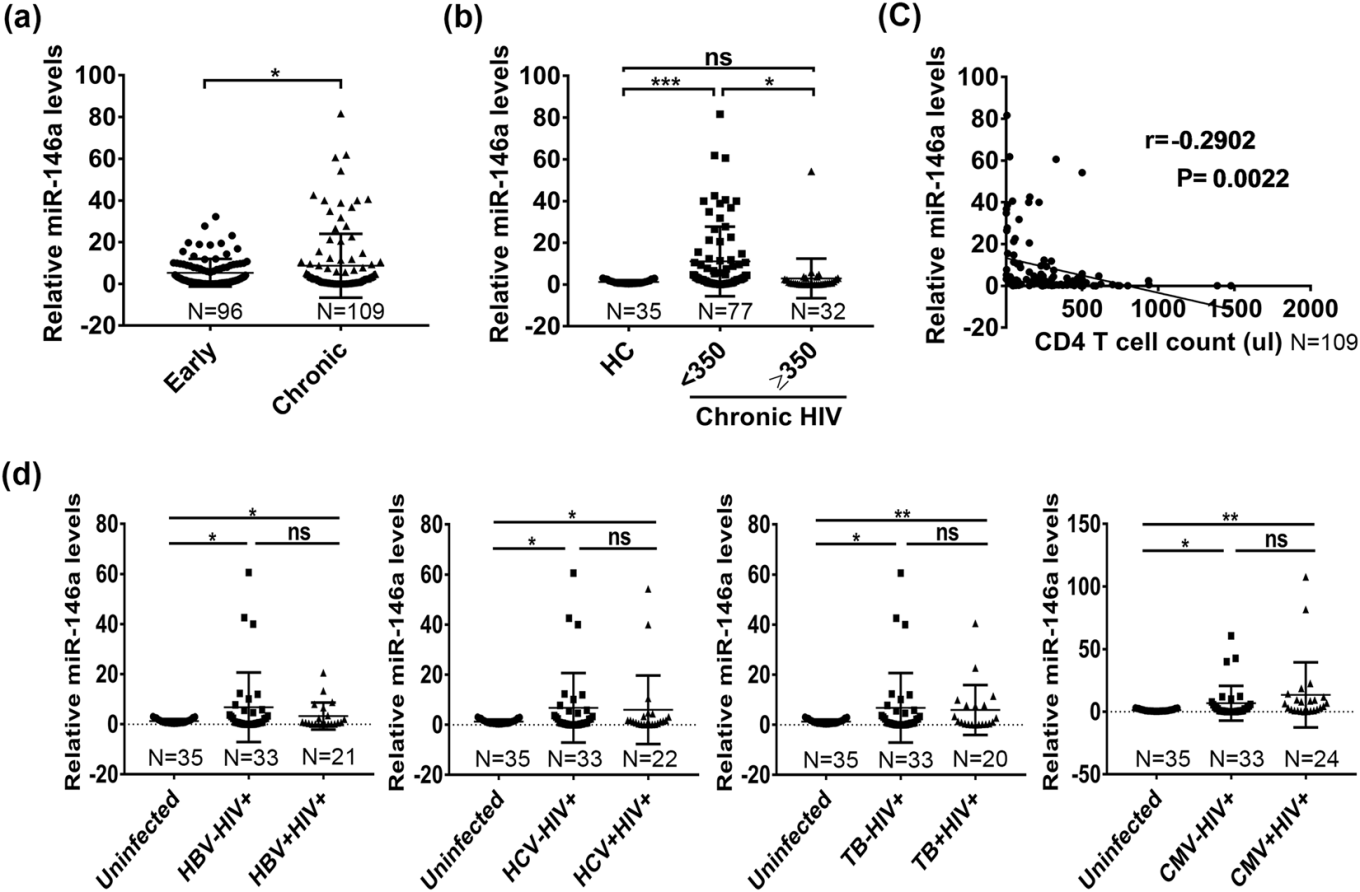
Changes in the expression of miR-146a in PBMCs from chronic HIV-1 infected patients. Quantitative PCR detection of miR-146a relative levels in PBMCs from early stage (n = 96) and chronic stage (n = 109) individuals of HIV-1 infected patients. (**a**) miR-146a relative levels in CD4+ T cell counts <350 cells/µl group (n = 77) were compared with CD4+ T cell counts ≥350 cells/µl group (n = 32) from chronic stage individuals or healthy controls (n = 35) (**b**), U6 was used as an endogenous control. (**c**) Correlation between miR-146a and CD4+ T cell counts was analyzed using Spearman’s correlation test (n = 109). Values of the correlative coefficient (r) and p are shown. (**d**) Quantitative PCR for miR-146a relative levels in the groups of HBV–HIV+ (n = 33), HBV + HIV+ (n = 21); HCV–HIV+ (n = 33), HCV + HIV+ (n = 22); TB–HIV+ (n = 33), TB + HIV+ (n = 20); CMV–HIV+ (n = 33), CMV + HIV+ (n = 24) and uninfected individuals (n = 35). *p < 0.05, **p < 0.01, ***p < 0.001. ns, not significant.

We next evaluated miR-146a levels in PBMCs from all HIV-1-infected individuals (Table 1, n = 205). Results showed that the levels of miR-146a in total PBMCs were higher in chronic HIV-1-infected patients than in early stage group and healthy controls (p < 0.05) (Fig. 2a,b). We also found that the miR-146a levels were higher in CD4+ cell counts <350 cells/µl group than CD4+ cell counts ≥350 cells/µl group (p < 0.05) (Fig. 2b).

We analyzed the correlations between miR-146a levels and CD4+ cell counts and found that miR-146a level was negatively correlated with the CD4+ cell count (r = −0.2902, P = 0.0022) (Fig. 2c). Positive correlations were also observed between miR-146a and CTLA-4/TIM-3 in chronic groups (Table 2).

**Table 2:** Correlation between miR-146a levels and immune exhaustion markers of PBMCs.

	HIV-1-positive subjects		
	Early stage (n = 27)	Chronic stage (n = 35)	All subjects (n = 62)
**miR-146a in PBMCs vs**			
CTLA-4	r = 0.038; p = 0.856	**r** = **0**.**408; p** = **0**.012*	**r** = **0**.**339; p** = **0**.007**
TIM-3	r = 0.306; p = 0.137	**r** = **0**.**423; p** = **0**.020*	**r** = **0**.**378; p** = **0**.003**
LAG-3	r = 0.186; p = 0.374	r = 0.076; p = 0.657	r = 0.091; p = 0.482

All correlations are reported as Spearman r and P values (two-tailed). Significant values are shown in bold. *p < 0.05, **p < 0.01.

Given that these chronic HIV-1-infected patients included some co-infected patients (Table 3), we therefore determined the expression of miR-146a in HBV/HIV, HCV/HIV, TB/HIV and CMV/HIV co-infected patients. We found levels of miR-146a did not differ upon co-infected or HIV-1-mono-infected patients. This suggested that co-infection might not increase miR-146a expression (Fig. 2d).

**Table 3:** General Characteristics of the Study Population.

Variable (n = 205)	Total
Age, years	45.3 (11.93)
Gender, male (%)	156 (76.1)
CD4 T lymphocyte count, cells/ml	196 (234.3)
Untreated for HIV-1, n (%)	94 (45.9)
HCV infection, n (%)	22 (10.7)
HBV infection, n (%)	31 (15.1)
Tuberculosis, n (%)	40 (19.5)
Cytomegalovirus, n (%)	40 (19.5)
Herpes Simplex Virus infection, n (%)	8 (3.9)
Oral Candidiasis, n (%)	39 (19)
Chronic Diarrhoea, n (%)	5 (2.4)

Categorical variables are expressed as frequencies (%). Continuous variables are expressed as mean (standard deviation). HC, Healthy control; ND, no data.

Taken together, chronic HIV-1 infection exhibited higher exhaustion markers as well as higher levels of miR-146a, which were positively correlated.

### Both HIV-1 infection and TCR stimulation induce miR-146a and exhaustion markers expression

**Figure 3:**
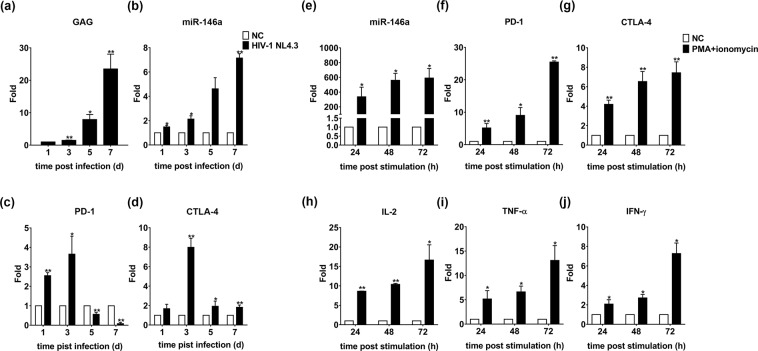
miR-146a is induced upon HIV-1 infection and TCR stimulation. Jurkat cells were infected with HIV-1_NL4-3_ (p24 750 ng/ml) for 3 h and washed three times in PBS, then cultured with fresh RPMI 1640 supplemented with 10% FBS for 1, 3, 5, and 7 days. The cells in each treatment group were then collected at the indicated time points. (**a**–**d**) Total RNA was isolated for quantitative PCR analysis of Gag, miR-146a, PD-1 and CTLA-4. Jurkat cells were stimulated with PMA and ionomycin, (**e**) miR-146a levels, exhaustion markers (**f**) PD-1 and (**g**) CTLA-4, and cytokines (**h**) IL-2, (**i**) TNF-α, (j) IFN-γ were detected at different time points by quantitative PCR. Each experiment was performed three times, and the results were shown as the mean fold change relative to control samples. *p < 0.05, **p < 0.01.

We observed continually increased miR-146a expression post HIV-1 infection in cell lines and primary monocyte-derived macrophages (MDMs) previously^20,24^. Herein, in HIV-1_NL4.3_ infected Jurkat cells, miR-146a levels were gradually increased during HIV-1 infection (Fig. 3a,b), and the mRNA level of PD-1 and CTLA-4 were increased to a peak in day 3, and then decreased (Fig. 3c,d).

However, we wondered how miR-146a was induced in chronic patients under suppressive antiretroviral therapy (ART) that successfully restricts the viral replication. Indeed, persistent immune activation is a central feature of HIV pathogenesis despite early/late initiation of ART^25^. The role of immune activation in the pathogenesis of non-AIDS clinical events (major causes of morbidity and mortality in people on antiretroviral therapy) received increasing concern^26^. miR-146a was reported to express during T cell differentiation responding to the activation of CD4+ T cells and CD8+ T cells^15,27^. Thus we used Jurkat T cells, a well-established model for *in vitro* study of TCR signaling, to investigate expressions of miR-146a and T cell genes. We first treated Jurkat cells with PMA and ionomycin, and then measured gene expression by RT-qPCR. As shown in Fig. 3e, miR-146a levels were significantly increased upon PMA and ionomycin stimulation, reaching a plateau after 48 hours. And the mRNA levels of exhaustion markers, such as PD-1 and CTLA-4, cytokines as IL-2, TNF-α and IFN-γ, were progressively increased upon PMA and ionomycin treatment (Fig. 3f–j).

These data demonstrate that not only HIV-1 infection but also T cell activation contributes to induction of both miR-146a and exhaustion molecules.

### miR-146a decreased antiviral cytokines production and the cytotoxicity of activated CD8+ T cells

**Figure 4:**
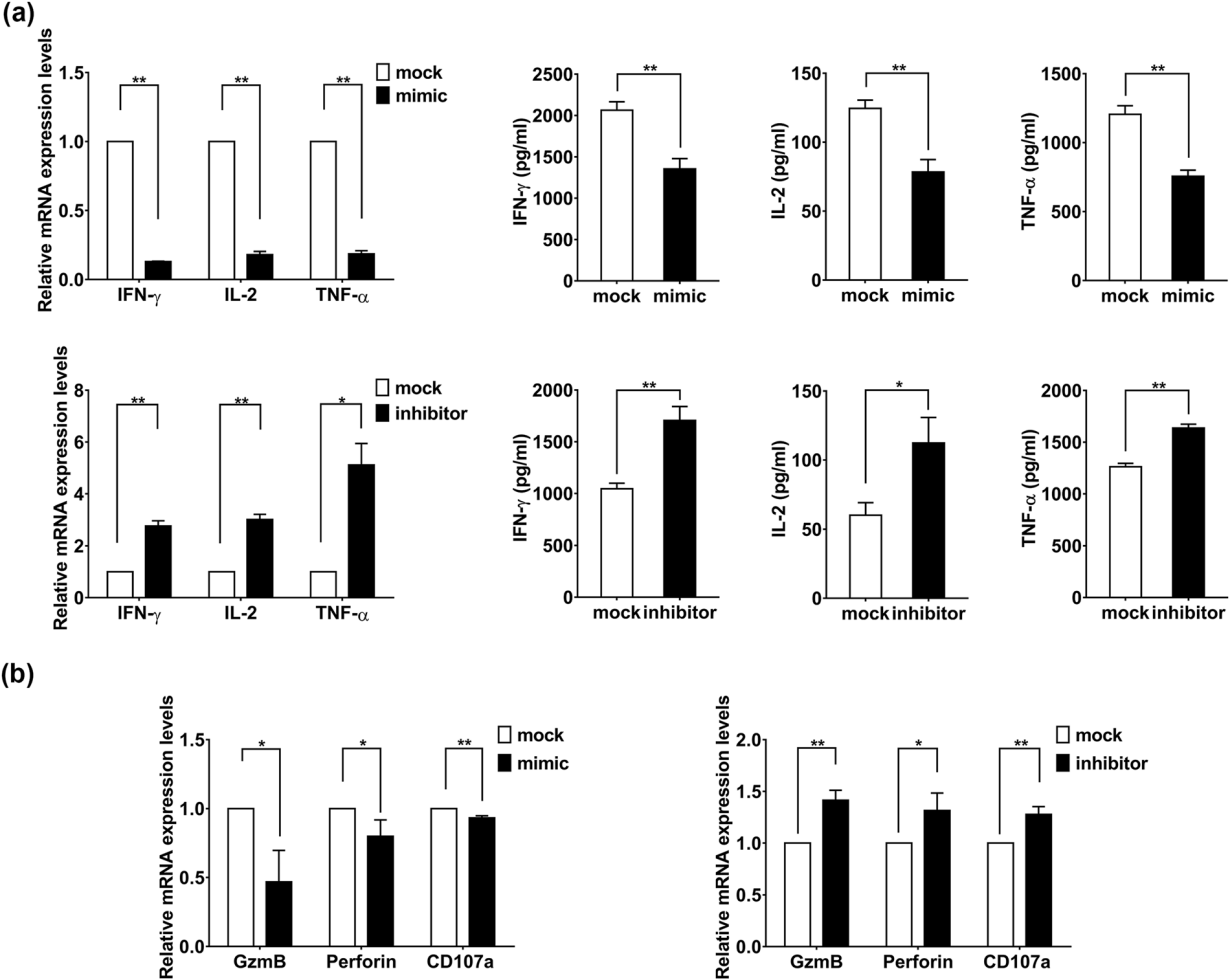
miR-146a reduces the production of antiviral cytokines and suppresses the function of T cells. CD8+ T cells from healthy individuals were transfected with 50 nmol/ml miR-146a mimic or miR-146a inhibitor, a randomized oligonucleotide served as a mock, and cultured in 1 mg/ml anti-CD3 for 48 h. (**a**) The relative mRNA levels of IFN-γ, IL-2, and TNF-α after transfected with miR-146a mimic and miR-146a inhibitor were assessed for real-time PCR using GAPDH as endogenous control. The levels of IFN-γ, IL-2, and TNF-α in the supernatant were detected by ELISA. (**b**) Quantitative PCR for GZMB, perforin and CD107a mRNA relative levels after transfected with miR-146a mimic or miR-146a inhibitor. Data shown as mean ± SEM. *p < 0.05, **p < 0.01.

To investigate the potential role of miR-146a on T cell function, we next examined anti-HIV cytokines production and the function state of human PBMC derived primary CD8+ T cells upon miR-146a overexpression. When CD3 antibody activated CD8+ T cells was transfected with a miR-146a mimic, significant decrease of IFN-γ, IL-2, and TNF-α were observed at both mRNA and protein levels, whereas miR-146a inhibitor greatly promoted the expressions of these cytokines (Fig. 4a). We also observed that mRNA level of GZMB and peforin were decreased when miR-146a was overexpressed and slightly increased when endogenous miR-146a was inhibited (Fig. 4b).

These data reveal that miR-146a may negatively regulate function of CD8+ T cells via decreasing antiviral cytokines production and alleviating the cellular cytotoxicity.

### *Ex vivo* neutralization of miR-146a improved the antiviral capacity of PBMCs from chronic HIV-1 infected patients

**Figure 5:**
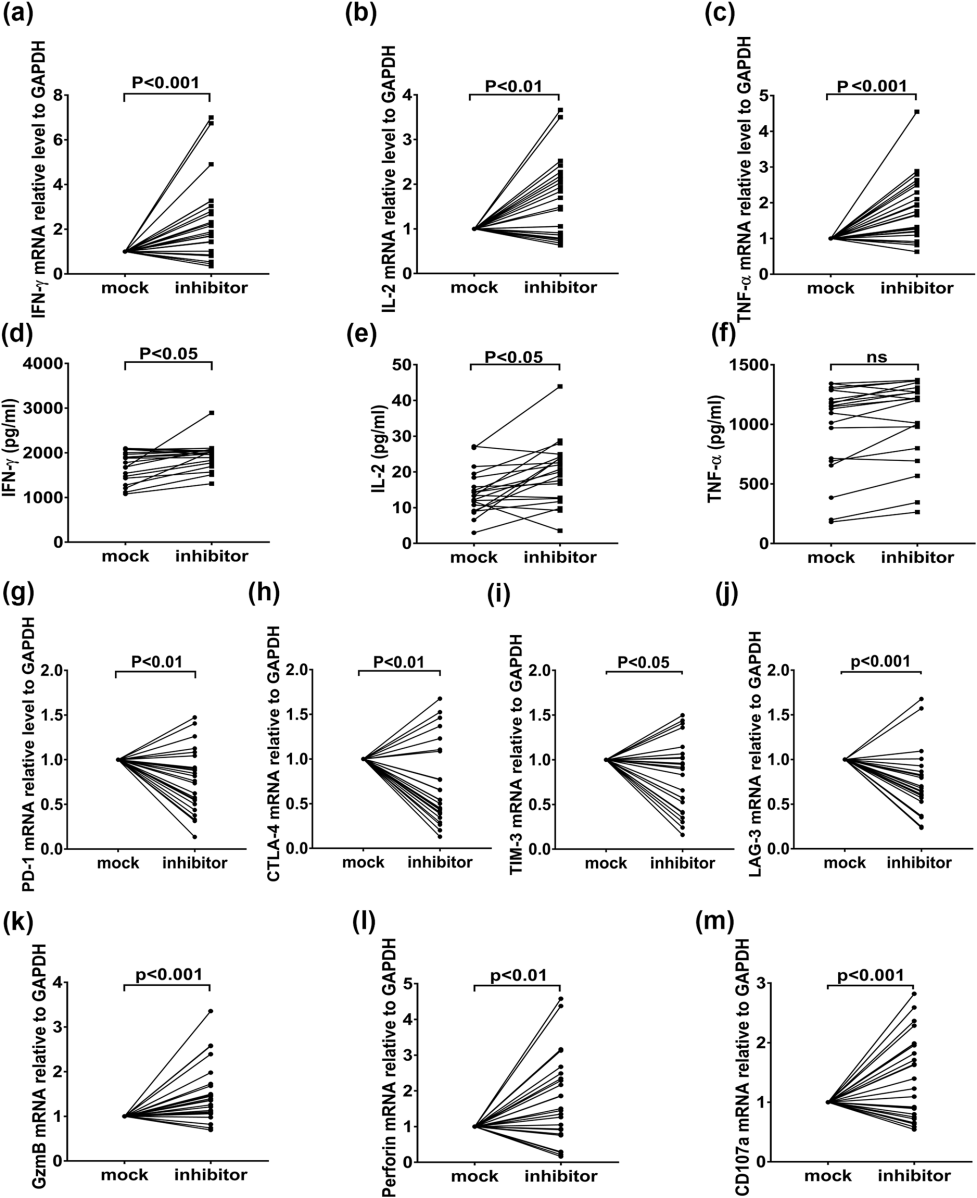
The blockage of miR-146a increases the antiviral genes production and decreased exhaustion markers in chronic HIV-1 infected patients. PBMCs from chronic HIV-1 infected patients (n = 24) were transfected with 50 nmol/ml miR-146a inhibitor or the randomized oligonucleotide as a mock. (**a**–**c**) Relative mRNA levels of IFN-γ, IL-2 and TNF-α in PBMCs from chronic HIV-1 patients were quantified by quantitative RT-PCR using GAPDH as internal controls. (**d**–**f**) The secretion of IFN-γ, IL-2 and TNF-α were detected by ELISA. Quantitative PCR detection of PD-1, CTLA-4, TIM-3 and LAG-3 mRNA relative levels (**g**–**j**) and CD107a, GZMB and perforin (**k**–**m**) mRNA relative levels in PBMCs from chronic HIV-1 patients, GAPDH was used as internal controls. Data shown as mean ± SEM.

Given that the miR-146a correlated positively with inhibitory receptors and negatively regulated T cell function, we next wondered whether elimination of miR-146a could restore the impaired T cell function from chronic HIV-1 infected patients. We transfected miR-146a inhibitor into PBMCs from 24 chronic HIV-1 infected patients and found that mRNA levels of antiviral cytokines, such as IFN-γ, IL-2 and TNF-α, had a significant increase (Fig. 5a–c). The protein levels of IFN-γ and IL-2 were consistently elevated (P < 0.05) (Fig. 5d,e), while the protein levels of TNF-α showed no significant difference (Fig. 5f). Simultaneously, levels of the inhibitory receptors showed a significant decrease (Fig. 5g–j). Moreover, levels of CD107a, GZMB and perforin were increased (Fig. 5k–m).

These data suggest that *ex vivo* blockage of miR-146a might reinvigorate the function of impaired immune cells from chronic HIV-1 patients.

### c-Fos levels in the peripheral blood of chronic HIV-1-infected patients were decreased and associated with miR-146a

**Figure 6:**
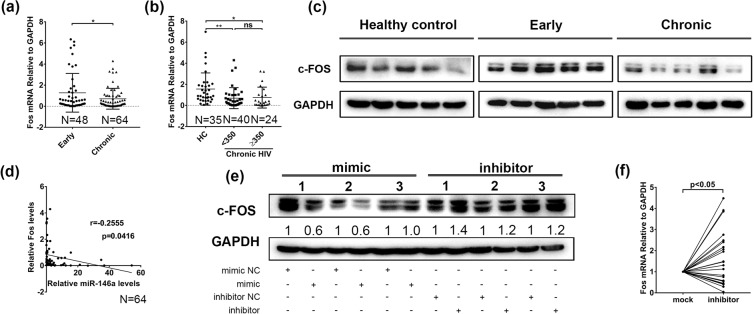
c-Fos expression was decreased in PBMCs from chronic HIV-1 infected patients. (**a**) Convincing data of c-Fos mRNA relative levels in PBMCs from early stage (n = 48) and chronic stage (n = 64) individuals of HIV-1 infected patients by quantitative PCR detection. (**b**) c-Fos mRNA levels in CD4+ T cell counts <350 cells/μl group (n = 40) and CD4+ T cell counts ≥350 cells/μl group (n = 24) from chronic stage individuals compared to healthy controls (n = 35), GAPDH was used as an endogenous control. (**c**) Western Blotting of c-Fos protein levels in PBMCs from healthy controls (n = 5), early stage (n = 5) and chronic stage (n = 5) HIV-1 individuals. (**d**) Correlation between c-Fos mRNA and miR-146a expression (relative) was analyzed using Spearman’s correlation test (n = 64). Values of the correlative coefficient (r) and p are shown. (**e**) CD8+ T cells from healthy individuals were transfected with 50 nmol/ml miR-146a mimics or miR-146a inhibitors for 48 h. c-Fos expression was measured by Western Blot. (**f**) PBMCs from chronic HIV-1 infected patients (n = 24) were transfected with 50 nmol/ml miR-146a inhibitor or the randomized oligonucleotide as a mock, levels of c-Fos mRNA in PBMCs from chronic HIV-1 patients were quantified by quantitative RT-PCR. *p < 0.05, **p < 0.01.

Previous study demonstrated that an engineered NFAT that could not cooperate with AP-1 strongly induced exhaustion^28^, which emphasized the role of AP-1 in CD8+ T cell exhaustion. And the AP-1 transcription factor subunit of c-Fos declined during chronic infection^28,29^. Hence, we evaluated c-Fos levels in PBMCs from chronic HIV-1-infected patients and healthy controls. The mRNA levels of c-Fos in total PBMCs were lower in chronic HIV-1-infected patients than in early stage group (p < 0.05) (Fig. 6a), and significantly lower than in healthy control group (p < 0.05) (Fig. 6b). We randomly selected PBMC samples from 5 healthy donors, 5 patients in early stage group and 5 patients in chronic stage group respectively to detect the protein levels of c-Fos, and found that c-Fos expression was down-regulated in chronic group (Fig. 6c). We further found that c-Fos mRNA level was negatively correlated with miR-146a in PBMCs from chronic HIV-1-infected patients (r = −0.2555; P = 0.0416) (Fig. 6d).

This inverse correlation suggested c-Fos as a potential target of miR-146a. We observed a slight decrease of c-Fos protein levels in primary CD8+ T cells after transfected with miR-146a mimic and a slight increase of c-Fos after miR-146a inhibitor transfection (Fig. 6e). We then wondered if neutralization of miR-146a would restore c-Fos expression in patients. After transfecting PBMCs from chronic HIV-1 infected patients with miR-146a inhibitor, we observed a significant increase of c-Fos mRNA (Fig. 6f), but no significant changes at protein level (Supplementary Fig. 1).

These data suggest that miR-146a might contribute to immune exhaustion partially through suppressing c-Fos, probably indirectly.

## Discussion

Cellular immune exhaustion has attracted increasing attention. Immune cells lose effect function and proliferation ability during chronic HIV infection. Recently, the functions of miRNAs in the immune response against viruses had attached attention and how miRNA expression may contribute to HIV-1 infection remains to be focused on. Previous studies have pointed out that the expression of miR-146a is lower in Elite Controllers as compared to Viremia Progressors^30^, which suggested that miR-146a may contribute to progression of HIV-1-infected patients. However, it remains unclear whether miR-146a is linked to cellular exhaustion in chronic HIV-1-infected individuals. Here in this study, we first found that miR-146a level was significantly higher in chronic HIV-1-infected group, especially when CD4+ T cells were under 350 cells/µl. As CD4+ T cell count is an important indicator that can reflect the status of cellular immune function, the negative correlation between miR-146a and CD4+ T cell counts suggests that miR-146a may be associated with the AIDS disease progression.

Immune exhaustion, typically defined by elevated expression of inhibitory molecules as PD-1, CTLA-4, TIM-3 and LAG-3^31^, is one of the hallmarks of HIV infection. We found that the PBMC levels of CTLA-4, TIM-3 and LAG-3 were higher in chronic HIV-1 infected patients, and showed a positive correlation with miR-146a relative level. But we failed to detect PD-1 mRNA in those freezing samples, although PD-1 is among the first reported inhibitory molecules related to immune exhaustion, typically up-regulated on HIV-1-specific CD8+ T cells and serves as a major regulator of apoptosis that have an effect on the frequency of antiviral T cells in HIV infection^31–33^. In addition, we also demonstrated that miR-146a could up-regulate the expression of exhaustion markers in many cell lines (data not shown). These findings suggested that miR-146a accumulated in chronic HIV-1 infection, positively correlated with exhaustion markers, and might intensify the cellular exhaustion.

We found that miR-146a could impair the antiviral response and cytotoxicity of CD8+ T cells. Moreover, blocking of miR-146a could recover the production of antiviral cytokines and ameliorate the exhausting state of immune cells in chronic HIV-1 infection. In other words, the blocking of miR-146a can partially restore the function of PBMCs in chronic HIV-1 infection. We performed the experiments on PBMCs, instead of derived T cells or other purified cell types, isolated from HIV-1 infected individuals and healthy controls, as the PBMCs stated in a mixed cell culture, could more accurately reflect what happen *in vivo*, to present in a more appropriate way about the state of exhaustion following cell-to-cell interaction during persistent HIV-1 infection.

We observed up-regulated miR-146a but down-regulated c-Fos in PBMCs from chronic HIV-1 patients. miR-146a mimic reduced while miR-146a inhibitor increased c-Fos proteins in human primary CD8+ T cells. In addition, c-Fos mRNA levels were significantly increased after miR-146a inhibitor treatment in 24 HIV-1 chronic patients’ PBMCs. However, we did not observed consistent c-Fos protein changes in PBMCs from 10 randomly selected HIV-1 patients under miR-146a inhibitor. This inconsistence indicated that other mechanisms might also be involved in c-Fos protein expression beyond regulation at c-Fos mRNA level. We could not define c-Fos as a direct target of miR-146a, at least based on the data provided here.

In conclusion, we discovered that PBMCs isolated from chronic HIV-1-infected patients had higher expression levels of miR-146a that were accompanied by a suppressed cellular cytotoxic activity. Our findings indicate that chronic HIV-1 infection and persistent immune activation may induce miR-146a expression, and the accumulation of miR-146a may subsequently result in the inhibition of the antiviral function of immune cells and lead to immune exhaustion. Therefore, miR-146a might be considered as an assistant predictor for immune exhaustion with potential to evaluate cellular immune functions in HIV/AIDS.

## Supplementary information

Supplementary information
