# Supplementary material for: Elevated expression of miR-146a correlates with high levels of immune cell exhaustion markers and suppresses cellular immune function in chronic HIV-1-infected patients
Source: Sci Rep. 2019 Dec 11;9:18829. doi: 10.1038/s41598-019-55100-2 (PMC6906421; doi:10.1038/s41598-019-55100-2)
Supplement: Supplementary file 1 — Supplementary information [file 41598_2019_55100_MOESM1_ESM.docx]

Supplementary information file

SREP-19-31840A

Title: Elevated expression of miR-146a correlates with high levels of immune cell exhaustion markers and suppresses cellular immune function in chronic HIV-1-infected patients

Author list: Ting Yu, Zhao Ju, Mingqi Luo, Ronghua Hu, Yan Teng, Linlin Xie, Chaojie Zhong, Lang Chen, Wei Hou, Yong Xiong, Yong Feng

**Content:**

**1. Supplementary Figure 1. WB of Fos in HIV-1 PBMCs after miR-146a inhibitor transfection.**

**2. Full length blot for Figure 6.**

1. Supplementary Figure 1. WB of Fos in HIV-1 PBMCs after miR-146a inhibitor transfection.


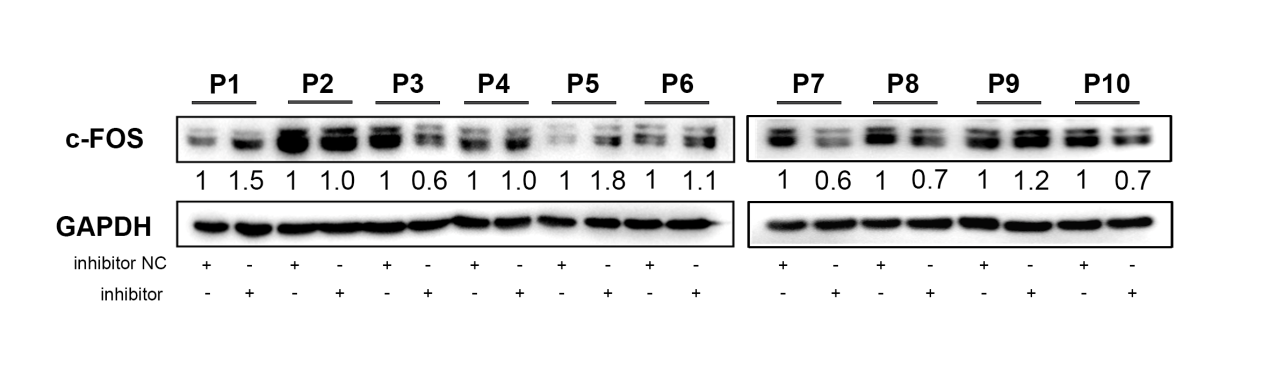


PBMCs from 10 HIV-1 infected patients were transfected with 50 nM miR-146a inhibitor or randomized oligonucleotide as a mock, the protein levels of Fos were detected by Western Blot 48 hrs post transfection. P1-P10 as patient 1-patient 10.

Full length blots for Supplementary Figure 1.


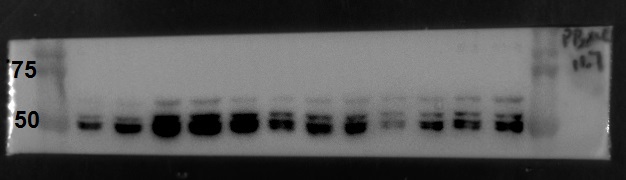
 c-FOS (P1-P6)


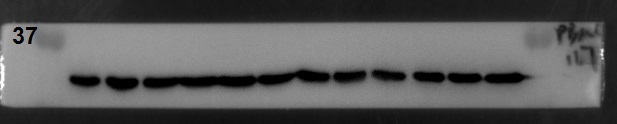
 GAPDH (P1-P6)


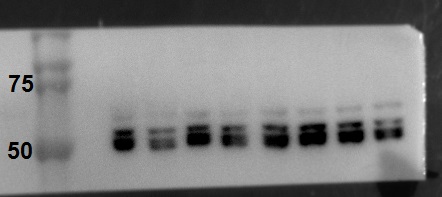
 c-FOS (P7-P10)


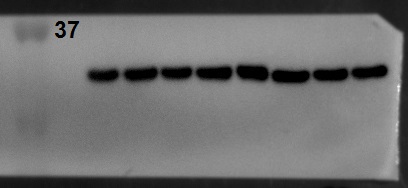
 GAPDH (P7-P10)

2. Figure 6


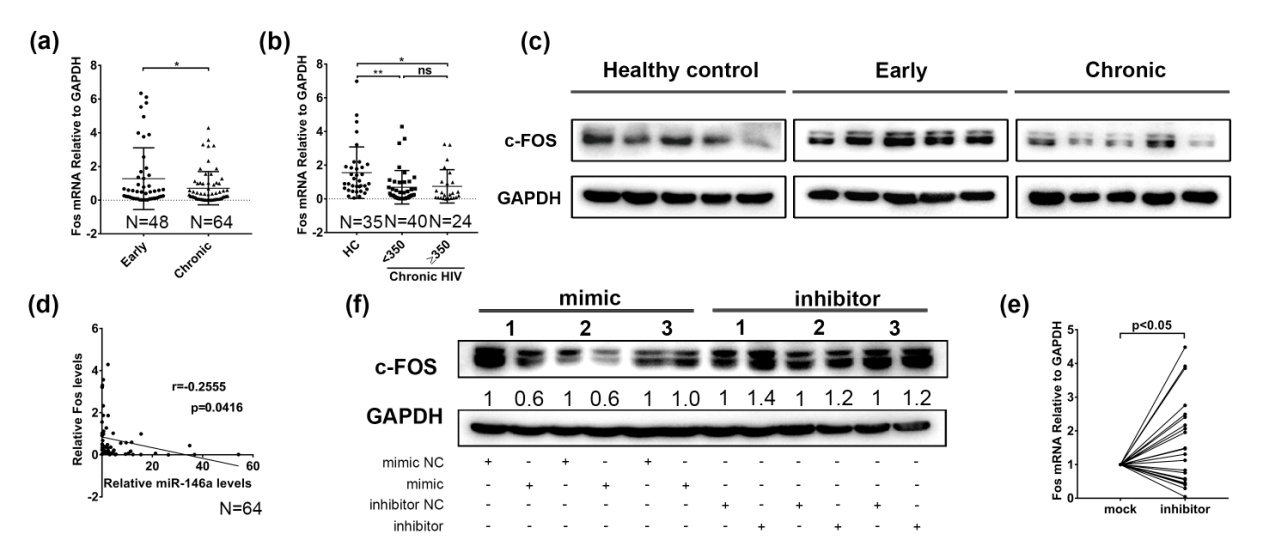


Full length blots of Figure 6c


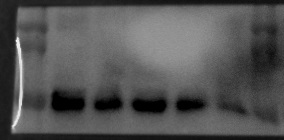
 c-FOS (Healthy control)


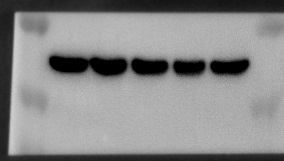
 GAPDH (Healthy control)


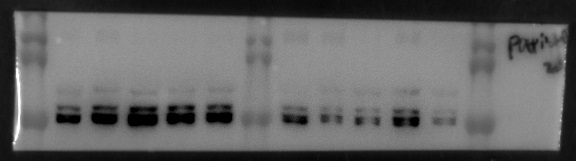


c-FOS (left 5 as Early, right 5 as Chronic)


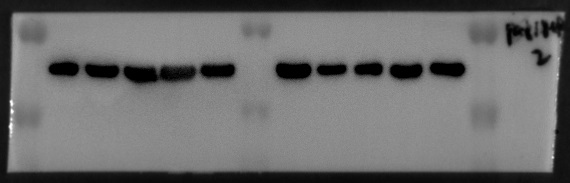


GAPDH (left 5 as Early, right 5 as Chronic)

Full length blots of Figure 6e


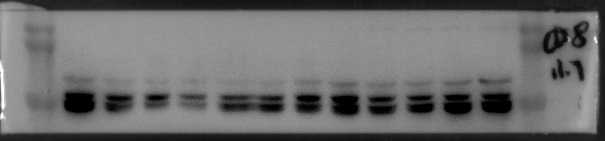
 c-Fos


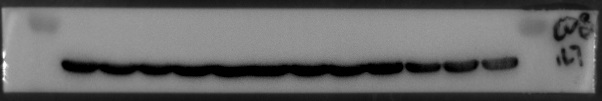
 GAPDH
